# Therapeutic factors and biomaterial-based delivery tools for degenerative intervertebral disc repair

**DOI:** 10.3389/fcell.2024.1286222

**Published:** 2024-02-05

**Authors:** Haoyang Song, Chuan Guo, Ye Wu, Yuheng Liu, Qingquan Kong, Yu Wang

**Affiliations:** Department of Orthopedic Surgery and Orthopedic Research Institute, West China Hospital, Sichuan University, Chengdu, Sichuan, China

**Keywords:** intervertebral disc, intervertebral disc degeneration, therapeutic factors, biomaterial, bioengineering therapeutics

## Abstract

Intervertebral disc degeneration (IDD) is the main cause of low back pain (LBP), which significantly impacts global wellbeing and contributes to global productivity declines. Conventional treatment approaches, encompassing conservative and surgical interventions, merely serve to postpone the advancement of IDD without offering a fundamental reversal. Consequently, there is an urgent demand for an effective approach to prevent the progression of IDD. Recent investigations focusing on the treatment of IDD utilizing diverse bioactive substances integrated within various biomaterials have exhibited promising outcomes. Various bioactive substances, encompassing conventional small molecule drugs, small molecule nucleic acids, and cell therapies, exhibit distinct capacities for repairing IDD. Additionally, various biological material delivery systems, such as nano micelles, microspheres, and hydrogels, possess diverse biological and release characteristics. Consequently, these diverse materials and drugs hold promise for advancing the treatment of IDD. This article aims to provide a concise overview of the IDD process and investigate the research advancements in biomaterials and bioactive substances for IDD treatment, delving into their mechanisms.

## 1 Introduction

Intervertebral disc degeneration (IDD) is a common cause of low back pain (LBP), which negatively impacts millions of people’s lives (Knezevic et al., 2021). According to reports, about 80% of the population suffers from LBP, which has been considered the most important cause of disability and has led to a decline in global productivity (Kazezian et al., 2020). Hence, it is imperative to promptly implement efficacious interventions aimed at mitigating IDD in order to address the resultant LBP, as it is widely believed that such interventions can enhance patients’ quality of life and minimize productivity losses. In the intervertebral disc (IVD), nucleus pulposus (NP) forms the center and annulus fibrosus (AF) surrounds it. These components connect two adjacent vertebrae through the cartilage endplates (CEP) at both ends, thereby offering cushioning and flexibility to the spine. IDD is a complex and continuous process in which multiple factors, such as genetics, aging, mechanical stress, and biochemical stimuli, collectively lead to an imbalance between the production and degradation of extracellular matrix (ECM), leading to aseptic inflammation. The increase in degradation and metabolism reduced the glycosaminoglycan in the NP, which disordered the structure of the ECM. Disruption of ECM metabolism led to changes in IVD mechanical properties and load-bearing capacity, resulting in secondary changes in AF and CEP. Moreover, ultimately, anatomical changes occurred, including decreased disc height, disc herniation, spinal stenosis, and nerve root compression. These anatomical changes were believed to be the main factors leading to LBP (Wu et al., 2022a).

At present, conservative and surgical treatment options are available for LBP. Common conservative treatments include physical therapy, exercise, and oral medication. However, these methods are usually aimed at solving pain symptoms and cannot fundamentally solve IDD. Surgical treatments such as spinal fusion have various potential adverse reactions, which can even lead to the worsening of adjacent segment IVD degeneration (Donnally et al., 2020). Therefore, the treatment of IVD NP degeneration using biomaterials as the basis and incorporating bioactive substances is receiving increasing attention as an emerging treatment option due to its potential to reverse IDD.

Different bioactive compounds have the ability to penetrate the NP by means of delivering biological materials, thereby modulating the microenvironment within the NP, rectifying ECM imbalance, and potentially reversing the IDD process. Traditional bioactive substances, such as cytokines, peptides, and drug molecules, can be incorporated or combined with biomaterials for delivery purposes. Additionally, small molecule nucleic acids, such as microRNAs (miRNAs) and small interfering RNAs (siRNAs), can specifically interact with biomaterials and enhance cellular and physiological activity by interfering with specific pathways within the NP. Moreover, stem cells’ ability to self-renew and differentiate into multiple lineages offers great hope for reconstructing impaired NP structures thanks to their regenerative capabilities. Consequently, the development of biomaterials for efficient cell delivery has emerged as a crucial area of research (Wang et al., 2022b).

In addition to biologically active substances used to improve IDD, biomaterial platforms for delivering these biologically active substances are also receiving attention. As a material with good biomechanical properties, hydrogels have good biocompatibility and the ability to carry and slowly release various drugs. In recent years, nanomaterials and micrometer materials have gradually gained attention. Delivery systems such as nano micelles, extracellular vesicles (EVs), microspheres, and hydrogels also play essential roles.

**FIGURE 1:**
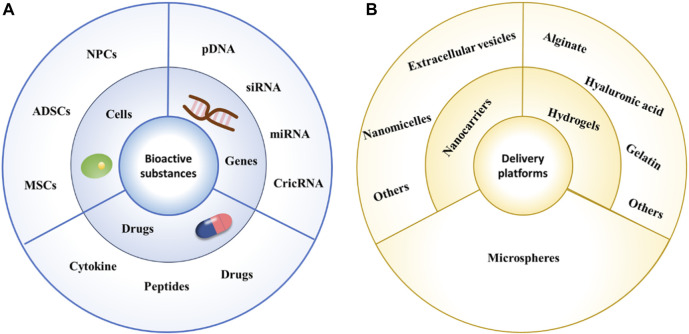
Schematic diagram of commonly used bioactive substances and delivery platforms. **(A)** Schematic diagram of commonly used bioactive substances. **(B)** Schematic diagram of commonly used delivery platforms.

In this review, we attempt to briefly describe the IVD structure and the process of degeneration and systematically elaborate on the latest developments in bioactive substances and their corresponding delivery systems used for IDD treatment, attempting to systematically and comprehensively present the latest research results (Figure 1).

## 2 IVD structure and degeneration

The IVD is situated between neighboring vertebral bodies within the spinal column and exhibits favorable load distribution properties, enabling it to effectively absorb and support the spinal pressure. IDD is associated with an imbalance between ECM production and degradation. Diminished tissue production and metabolism, coupled with heightened degradation metabolism, result in a decline in glycosaminoglycan levels within the NP and structural irregularities within the ECM. These factors may subsequently contribute to intervertebral disc herniation and the onset of LBP. The IVD comprises the gel-like NP located at the center, the elastic AF surrounding it, and the CEP connecting it to the adjacent vertebrae. The IVD lacks blood vessels and innervation, with only a limited presence of blood vessels and nerves in the AF (Urban and Roberts, 2003). Consequently, the nutrition of the IVD primarily depends on diffusion through the cartilage endplate (Rajasekaran et al., 2004). This characteristic not only hampers the regenerative capacity of the IVD but also poses challenges in terms of external drug-based treatments.

It is widely accepted that the origin of NP can be traced back to the notochord, and the presence of notochord cells in the IVD of children is evident until the age of 10. Subsequently, a gradual replacement of notochord cells occurs, wherein mature NPCs with diminished metabolic activity take their place (Boos et al., 2002). NP ECM is rich in type II collagen, proteoglycans, and hyaluronic acid (HA). The presence of proteoglycan and HA maintains the high osmotic pressure of the NP, helps the NP perform normal physiological functions, carries pressure, and reduces deformation.

AF is composed of type I collagen with a consistent direction, forming a series of concentric circular structures and providing good compressive and tensile capabilities (Peredo et al., 2021). The inner layer of the AF is mainly composed of type I collagen with poor directionality, and its biomechanical properties are not prominent, while the outer layer of the AF is denser and more uniform, with good tensile properties (Takimoto et al., 2019). The CEP connects the IVD with the vertebral body, mainly composed of hyaline cartilage. The limited vascularization of the IVD necessitates the predominant entry of nutrients and other substances through the CEP, thereby establishing the CEP’s crucial involvement in maintaining regular nutritional supply to the IVD. Furthermore, owing to its cartilaginous composition, the CEP also assumes responsibility for the transmission and distribution of pressure during physical activity (Liu et al., 2023).

Multiple factors, such as genetics, aging, mechanical stress, and biochemical stimuli, cause IDD. IDD often starts in young people and gradually worsens with age and various factors. The pathophysiology of IDD can be divided into three stages: early, mild, and severe.

The early degeneration of IVD is often related to the content of ECM in the NP. With the decline of notochord cells and the change in NPC phenotype, type II collagen and proteoglycan synthesis decrease and type II collagen is gradually replaced by type I collagen, causing a decrease in IVD height and load capacity (Kirnaz et al., 2022; Mahyudin et al., 2022). At the same time, NPCs produce abnormal amounts of matrix metalloproteinase-1 (MMP-1) and MMP-3 as well as a metalloprotease with thrombospondin motifs (ADAMTS) with a platelet reactive element motif, which inhibits the synthesis of proteoglycan and collagen II, mediate the degradation of ECM, and jointly lead to the disorder of ECM metabolism. The reduction of proteoglycans plays the most critical role in early metamorphosis, mainly manifested by the loss of aggregated proteoglycans and the transition from chondroitin sulfate to keratin sulfate, leading to an increase in other ECM components.

At this stage, the IDD process is often mild and unnoticed, with a weaker degree of degeneration and easier repair. Therefore, early degeneration related to NP organization is considered by many to be the core of changing the IDD process, and many bioactive substances and delivery systems are also designed for early NP repair.

To summarize, the primary factor in IDD repair is the early degeneration associated with NP organization. Subsequently, our attention will be directed towards NP repair, wherein we will thoroughly and systematically examine the employment of bioactive substances and delivery systems, thereby presenting the most recent research findings in a comprehensive manner.

## 3 Therapeutic factors

Due to the complexity of the IDD progression, to accomplish IVD regeneration and repair, various bioactive substances have been explored for the suspension and reversal of IDD development, including but not limited to cellular therapy, genetic therapy, small molecule drugs, other agents, and combination drug delivery systems. These substances have different mechanisms of action and play a role in different histological characteristics during the IDD process. Overall, they can partially fill damaged NP tissue, alleviate the inflammatory environment in the NP, correct disordered ECM metabolism, and thus restore the structural and mechanical properties of the IVD.

### 3.1 Cells

In recent years, delivering various live cells into NP to fill damaged tissue and reverse the IDD process has received widespread attention. *In situ* nucleus pulposus cells from IVD, and various MSCs from different tissues have been used in IDD animal models to evaluate their efficacy in repairing IDD and demonstrate promising results.

#### 3.1.1 Mesenchymal stem cells-based IDD regeneration

MSCs are pluripotent stem cells that can self-replicate. Under certain conditions, these cells can differentiate into various functional cells, including NP-like cells, which is of great significance for the repair of IDD. In constructing repair materials for IDD, we can induce its differentiation into NP-like cells through various methods, such as active cellular substances, hypoxia, genetic engineering, and mechanical stimulation, to reconstruct NP structure and restore normal IVD function.

**FIGURE 2:**
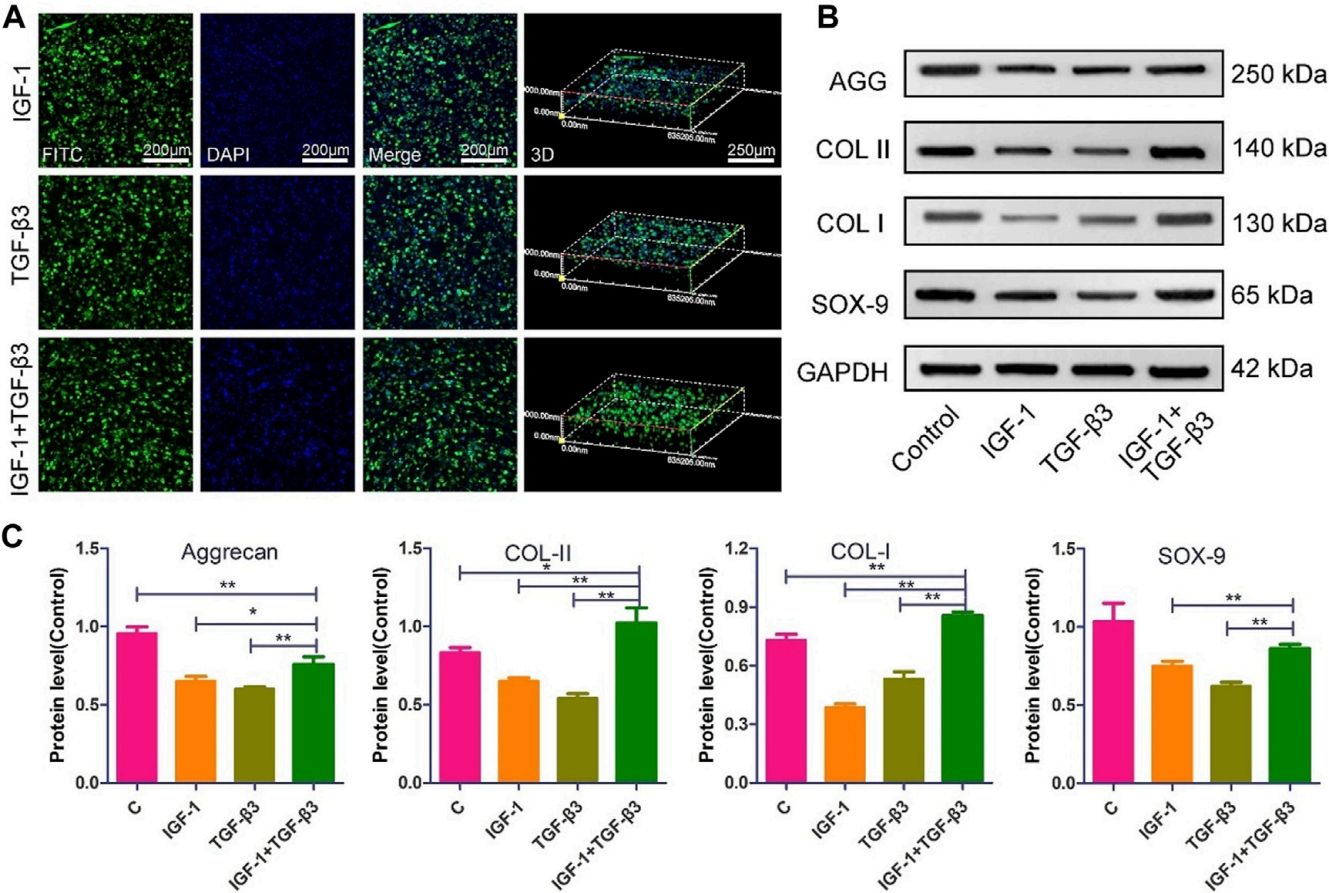
The morphology and ECM expression of NP MSCs following gene transfection (Ye et al., 2023). **(A)** Observations of the morphological characteristics of NP-MSCs following transfection within the hydrogel. **(B)** Quantification of protein expression levels for the transfected cells within the hydrogel across the three groups. **(C)** Measurement and analysis of protein expression levels in the transfected cells from the three groups, performed through quantitative methods. (n = 3, * and * * indicate *p* < 0.05 and *p* < 0.01 respectively).

The NP repair strategy based on MSCs has been extensively validated. Feng et al. (2011) combined a hypoxic environment with transforming growth factor (TGF)-β3, rabbit MSCs were successfully induced to differentiate into NP-like cells, nucleus pulposus-related genes (aggrecan, type II collagen, and Sox-9) were upregulated and high levels of HIF-1α expression were also detected, which may play a protective role against IVD degeneration, and their phenotype was stable subcutaneously in rats. Furthermore, Zeng et al. (2015); Wang et al. (2021b) delivered canine MSCs and rabbit MSCs into corresponding animal models. This strategy based on delivering MSCs into NP and differentiating them into NP-like cells has been observed to inhibit and reverse IDD in both rabbit and dog models, providing evidence and hope for treating IDD with MSCs. With the increasing maturity of genetic engineering technology, Ye et al. (2023) further utilized lentivirus-mediated insulin-like growth factor-1 and TGF-β3 dual gene transfection to effectively promote the proliferation and differentiation of NP MSCs into NP-like cells *in vitro*, increasing the effectiveness of MSCs in IDD treatment and proving the potential therapeutic value of genetic technology in IVD regeneration (Figure 2).

Recently, Adipose-derived stem cells (ADSCs) have also played a role in treating IDD. Ying et al. (2022) used N-propionyl mannosamine to develop and modify ADSCs through metabolic sugar engineering, regulate cell biological functions, promote cell ECM adhesion, and guide NP-like differentiation, providing new insights into ADSCs’ clinical application in IDD treatment. Huang et al. (2023) utilized the mechanical response characteristics of ADSCs to stimulate their differentiation into NP-like cells successfully. Simultaneously, increasing the ECM and water content in IDD in the body, reducing the height loss of degenerative IVD, and partially restoring the mechanical properties of degenerative IVD provides enormous potential for regulating stem cell differentiation in IVD repair.

#### 3.1.2 Other cell-based IVD regeneration

In addition to MSCs, cells derived from the IVD, such as NPCs and AF cells, are also highly valued. Although these cells have lower proliferation ability than MSCs, they may have more significant potential in reconstructing damaged IVD structures than cell-less delivery systems.

Barcellona et al. (2021) used a hydrogel system to deliver NPCs to treat IDD. Similarly, in another study, Gan et al. (2017) delivered NPCs into NP through carriers. Both strategies effectively promote rehydration and regeneration of degenerative NP, providing a reference for treating NPCs in IDD.

**FIGURE 3:**
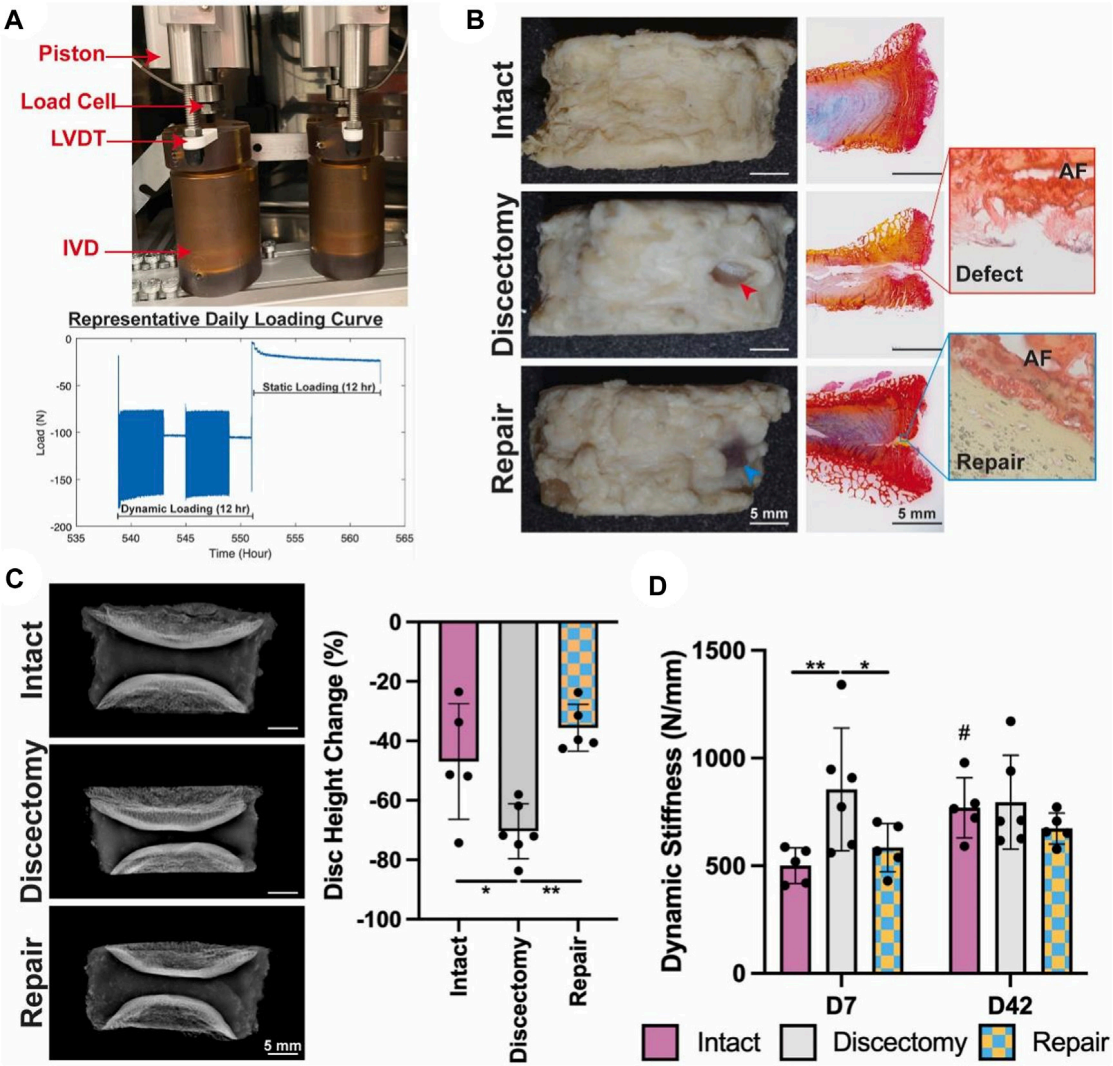
The application of composites for repair prevents injury-induced disc height loss and acute stiffening of the IVD, after a 42-days *ex vivo* organ culture (Panebianco et al., 2022). **(A)** The Loading System for Disc Organ Culture (LODOC) bioreactor, along with a representative daily loading curve. The continuous measurement of disc height was conducted using a linear variable differential transformer (LVDT). **(B)** Day 42 images (left) paired with picrosirius red/Alcian blue histology (right). Red and blue arrowheads are used to denote injury and repair, respectively. **(C)** X-ray images on Day 0 (left) and the percentage change in disc height on Day 42 (right). **(D)** Dynamic stiffness was determined from the LODOC load and displacement data at Day 7 and Day 42.

Although the NP is usually the core organization of the IDD process, as IDD worsens, AF tissue also undergoes damage. Therefore, AF cells’ mid to late-stage IDD repair is also highly valued in addition to NPCs. Based on this, Panebianco et al. (2022) designed a complex multilevel delivery system to deliver AF cells, successfully synthesizing a large number of ECM *in vitro* organs and restoring the biomechanical properties of IVDs (Figure 3).

In summary, the cell-based IDD repair strategy has a relatively ideal effect, as it addresses the issues of cell apoptosis and phenotypic changes during the IDD process. However, due to the large volume of cells themselves, direct delivery will inevitably lead to tissue damage and leakage. The most critical issue is designing a delivery system with good biocompatibility and biomechanical properties. Furthermore, when this strategy is applied to the human body, the immune interactions between cells from other sources and the treatment recipient are also worthy of attention.

### 3.2 Genes

Abnormal gene expression is usually the root cause of IDD. With the maturity of genetic technology, various nucleic acids, such as plasmid DNA (pDNA), siRNAs, miRNAs, and circular RNA (circRNA), have been found to have different mechanisms of action and can regulate the level of cell gene expression. In recent years, gene therapy that directly introduces these nucleic acids into IVD through delivery systems has also shown promise for treating IDD.

#### 3.2.1 pDNA

pDNA is a small circular DNA molecule originally derived from bacteria. Its simple structure, convenient construction, autonomous replication ability, and low cost make it a promising therapeutic strategy. Feng et al. (2015) selected the pDNA that induces the expression of heme oxygenase-1. The results indicated that the expression of heme oxygenase-1 significantly reduced the inflammatory response induced by interleukin (IL)-1β, increased the production of NP extracellular matrix, and significantly slowed the progression of IDD, which has excellent potential for NP regeneration. On this basis, Feng et al. (2017) further selected NR4A1 (a potential novel therapeutic target for fibrosis) pDNA as the active substance for treating IDD, significantly reversing fibrosis and promoting IVD regeneration in a rat tail model. However, its mechanism still needs further exploration. Due to its relatively large size, the design of its delivery system is further required.

#### 3.2.2 siRNAs

siRNAs can silence specific genes after transcription by degrading corresponding messenger RNA (mRNAs), and based on this characteristic, many studies have identified it as a potential drug. However, the degradation of simple siRNAs by ribonucleases *in vivo*, their limited half-life, the necessity for multiple repeated injections to attain therapeutic effects, the consequent heightened risk to patients, and their inability to independently traverse the cell membrane due to their negative charge, all contribute to suboptimal transfection efficiency and therapeutic efficacy. Chen et al. (2022) demonstrated that interferon gene stimulator (STING) acts as a DNA sensor to induce nuclear factor kappa-light-chain-enhancer of activated B cells (NF-κB)-dependent inflammation and degeneration, effectively transports siSTING in the IVD and continuously silences the expression of STING in NPCs. In another study, Chen et al. (2023a) also selected siRNAs based on targeted inhibition of P65 protein expression to silence the P65/NLRP3 pathway, successfully inhibiting the inflammatory microenvironment, significantly delaying the IDD process and combining it with cell therapy, successfully achieving IVD regeneration and repair.

#### 3.2.3 miRNAs

MiRNAs, a group of diminutive endogenous RNAs devoid of coding capacity, participate in the downregulation of gene expression through their interaction with target mRNAs. Therefore, specific miRNAs may also be able to achieve the goal of treating IDD by regulating the expression of some genes that play specific functions in the process of IDD. Feng et al. (2018) selected miR-29a in the miRNA-29 (miR-29) family, which has been proven to have the ability to inhibit fibrosis, and sent miR-29a into the IVD, effectively silencing the expression of MMP-2, significantly slowing down IVD fibrosis, and achieving MMP inhibition. In another study, Ji et al. (2018) selected miRNA-141, the expression of which is associated with IDD grading and has been evidenced to be indispensable in the pathogenesis of IDD. MiRNA-141 was delivered through nanoparticles, significantly inhibiting the progression of IDD in the IDD model.

**FIGURE 4:**
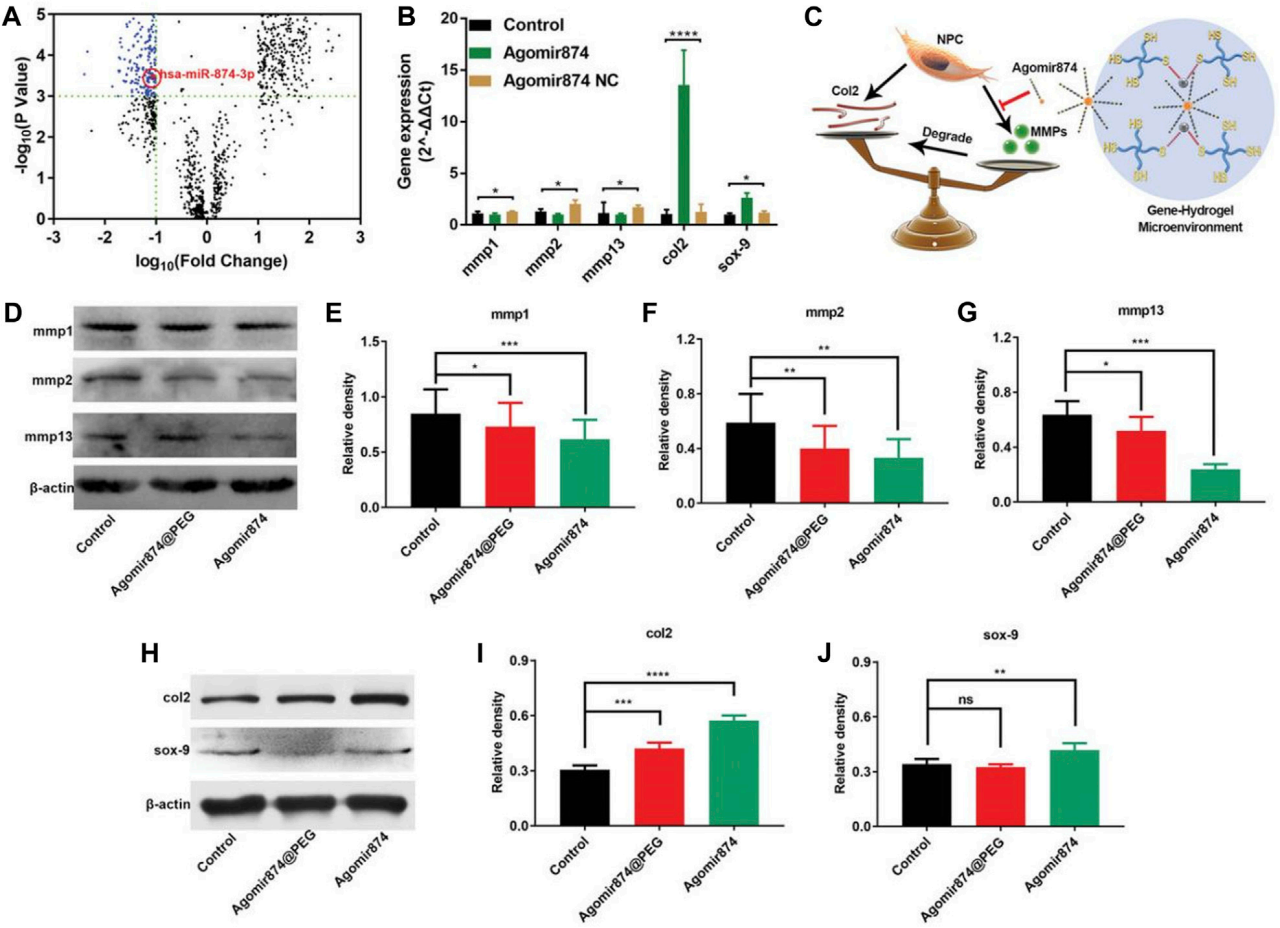
Agomir874@PEG’s impact on regulating the synthesis/catabolism balance of NPCs *in vitro* (Chen et al., 2020). **(A)** The volcano plot illustrates the differential expression of miRNAs in IDD. **(B)** Polymerase chain reaction data showcasing the gene expression in NPCs treated with Agomir874 or Agomir874 negative control. **(C)** A diagram illustrating the effects of the gene-hydrogel microenvironment on regulating the balance between synthesis and catabolism. **(D)** Western blotting data demonstrating the down-regulation of MMP expression. Specifically, the levels of **(E)** MMP-1, **(F)** MMP-2, and **(G)** MMP-13 are depicted in the data. **(H)** Western blotting data depicting the up-regulation of expression levels for type II collagen and Sox-9. Specifically, the levels of **(I)** Type II collagen and **(J)** Sox-9 are presented in the data.

Nevertheless, the utilization of miRNAs for treatment is hindered by their susceptibility to degradation and instability. However, the therapeutic efficacy can be enhanced by employing cholesterol-modified miRNAs (agomir) or inhibitors (antiagomir), which exhibit similar mechanisms of action to miRNAs.Based on this, Chen et al. (2020) selected agomir-874 based on the miRNA-874 structure and successfully downregulated the expression of MMP, balancing the metabolism of the ECM within the NP, providing opportunities for IVD regeneration, and achieving the therapeutic effect of IDD (Figure 4).

In addition, in the use of antagomir, Chen et al. (2023b) found that antagomirs based on miR-204-3p can inhibit the apoptosis of NPCs and designed a hydrogel for delivery, showing good IVD recovery, water retention, and structural maintenance. Wang et al. (2022b); Wang et al. (2023a); Wang et al. (2023b) were concerned about the role of miRNA-21 in IDD in NPC abnormal proliferation and ECM degradation, so they chose antagomir-21 to selectively inhibit miRNA-21, achieving the effect of regulating ECM metabolic balance through the inhibition of the MAPK/ERK signaling pathway, delaying the progression of IDD and achieving IDD repair.

#### 3.2.4 CircRNA

CircRNA is a type of large endogenous noncoding RNA, with its 5′and 3′terminals interconnected to form a closed-loop structure, making its expression more stable and less prone to degradation. In recent years, research has shown that circRNA is rich in miRNAs binding sites and can adsorb a large amount of miRNAs in cells, acting as a miRNAs sponge, thereby easing the inhibitory influence of miRNAs on its target gene, leading to an augmentation in the expression level of the target gene. Therefore, the role of circRNA in gene therapy for the IDD process is also receiving increasing attention. Cheng et al. (2018) found that circVMA21 can adsorb miRNA-200c, reduce the expression of its target X-linked inhibitor of apoptosis protein (XIAP), and successfully alleviate the degenerative changes of NP by injecting circVMA21 into a rat IDD model. Du et al. (2022) found that the expression of circ_0083756 exhibited a significant rise in degenerative NP tissues and cells, adsorbing miRNA-558, promoting the process of IVD, and alleviating IDD in a rat model by silencing circ-83756. It should be noted that although some circRNA have different functions in regulating IVDD, the role and underlying mechanisms of circRNA in IVDD are still largely vague and require further research.

In summary, many different types of gene therapy strategies have been attempted for the treatment of IDD, each with its advantages and disadvantages. However, overall, our understanding of genes is still insufficient, and the underlying mechanisms and potential risks still need to be studied. Therefore, finding a suitable delivery carrier that can effectively leverage strengths and avoid weaknesses, solve safety issues, and fully utilize therapeutic effects has become a bottleneck problem in gene delivery.

### 3.3 Small-molecule drugs

Small molecule drugs are typically characterized by their ease of synthesis, theoretical maturity, and extensive utilization in various systemic diseases. The treatment of IDD has also seen the exploration of numerous small molecule drugs, encompassing cytokines, peptides, and conventional drugs. The impact of small molecule drugs on the IDD process is multifaceted, with distinct mechanisms of action observed among different drug types, such as anti-inflammatory, anti-apoptotic, antioxidant, and IDD-blocking effects. Furthermore, certain small molecule drugs exhibit synthesis and anti-decomposition properties, which significantly influence their efficacy.

#### 3.3.1 Cytokine

TGF-β3, a crucial member within the TGF-β superfamily, is responsible for the regulation of fundamental biological processes, including proliferation, survival, and differentiation. Its role in promoting NP tissue formation, cartilage repair, and accelerating chondrogenic differentiation has been widely recognized. Therefore, TGF-β3 in IDD repair has received widespread attention. Zhu et al. (2022) prepared hollow mesoporous MnO_2_-loaded TGF-β3 nanoparticles that significantly reduced type II collagen degradation and iNOS expression in a rat IDD model. In another study, Ligorio et al. (2021) designed an injectable graphene oxide (GO)-self-assembling peptide FEFKFEFK (F: phenylalanine; K: lysine; E: glutamic acid) hybrid hydrogel as a TGF-β3 delivery system, and ECM reconstruction was found in in vitro experiments.

In addition, Zheng et al. (2019) paid attention to the role of transcription factor EB (TFEB) in inhibiting the excessive apoptosis and aging of NPCs through the ganglion autophagy-lysosome pathway. They demonstrated its role in improving IDD by overexpressing TFEB in a rat IDD model through lentivirus transfection. Therefore, TFEB may also be a potential target for IDD treatment. In recent years, treating inflammatory factors with antagonists has also been a focus of attention. Xu et al. (2020b) used recombinant human soluble tumor necrosis factor (TNF) receptor type II (rhsTNFRII), successfully inhibiting their denaturation by inhibiting inflammatory cascade reactions and regulating ECM synthesis/metabolic balance. However, due to the polymorphisms of cytokine action and toxicity due to activation of non-target cells, whether cytokines leaking from the disc poses a potential risk still needs further investigation.

#### 3.3.2 Peptides

Peptides, which are bioactive compounds, play a crucial role in diverse cellular functions and serve as the fundamental building blocks of proteins. Notably, the utilization of bioactive peptides for the regulation of IVD cells and enhancement of IDD processes has garnered growing interest in recent times. Tang et al. (2022) delivered three short peptide fragments, SNVI, KPSS, and KAIS, which have the biological activity of bone morphogenetic protein-7. They found that KPSS exhibited the most potent biological activity and most extended duration of action. Based on the peptide strategy, Bian et al. (2021) selected the bioactive peptide APETx2 and further carried the nucleus pulposus. Through both *in vitro* and *in vivo* experiments, this strategy downregulated the expression of acid-sensitive ion channel-3, effectively suppressing the inflammatory response caused by IDD and fostering the proliferation of nucleus pulposus cells, along with the regeneration of ECM.

#### 3.3.3 Drugs

A diverse range of drugs exists, each with distinct side effects, which can effectively regenerate IVD cells by mitigating inflammation and cellular damage, as well as stimulating synthetic metabolism. Consequently, this process aids in alleviating the progression of IDD. Given the sterile inflammatory environment within degenerative NP, conventional nonsteroidal anti-inflammatory drugs can impact the arachidonic acid cascade reaction, leading to a reduction in inflammation and pain. Teixeira et al. (2016) used diclofenac, which reduced the production of IL-6, IL-8, and prostaglandin E2, downregulated the expression of MMP-1 and 3, and upregulated the production of type II collagen and aggrecan.

Chen et al. (2016) proved through *in vivo* and *in vitro* settings that metformin therapy induces autophagy in nucleus pulposus cells in an Adenosine Monophosphate-Activated Protein Kinase dependent manner, thereby exerting anti-apoptosis and anti-aging effects of antioxidant stress. On this basis, Zheng et al. (2022) used metformin to successfully reverse the damage of high glucose to the ECM *in vitro*/*in vivo*. These findings indicate the therapeutic potential of metformin in preventing IDD, especially in patients with diabetes. Bai et al. (2019) paid attention to the role of rapamycin in inducing autophagy, thus protecting the cartilage extracellular matrix from degradation, and used rapamycin to modulate the local inflammatory microenvironment of IDD and facilitate the regeneration of intervertebral disc IVD tissue and proved that it clears reactive oxygen species (ROS) and induces M2-like macrophages, thereby diminishing the inflammatory response and supporting the regeneration of the rat IVD.

Lim et al. (2022) delivered ABT263, a senolytic drug that selectively induces apoptosis of aging cells by destroying the interaction between Bcl-2/Bcl-xL and pro-death proteins, into IVD, significantly arresting the development of IDD and reinstating the structural integrity of the IVD in a rat model. Berberine has anti-inflammatory, antioxidant, anti-apoptosis, and other activities. Chen et al. (2018) found that Berberine can inhibit the apoptosis of NPCs induced by oxidative stress through autophagy activation, thus reducing the ECM degradation of NPCs under stress. Novais et al. (2021) used senolytic drugs combined with dasatinib and quercetin for IDD treatment and proved that they can reduce local aging status, fibrosis, and matrix degradation.

#### 3.3.4 Others

As the comprehension of pharmaceuticals continues to advance, a plethora of unconventional bioactive compounds have been identified and employed in the therapeutic management of diverse ailments. Zhou et al. (2022) demonstrated that Prussian blue nanoparticles (PBNPs) have the capacity to enhance the function of intracellular mitochondria, elevate antioxidant capacity both inside and outside NPCs, and uphold cells in a reducing environment. It results in an improvement in the cell’s anabolic capacity and contributes to the reconstruction of the ECM in the NP, thereby providing a treatment for reactive ROS-mediated IVD degeneration in animal models. Similarly, Yang et al. (2022) also successfully reduced the production of ROS in NPCs through PBNPs, restored the height of IDD, reduced the decrease in water content, and reversed IDD. In addition to PBNPs, other anti-ROS drugs are also used for the treatment of IDD.

Tao et al. (2022) used coumarin-based NO donors to achieve controlled release of NO, exerting antibacterial, anti-inflammatory, and anti-fracture effects, effectively treating IDD related to Modic changes in rat models. Li et al. (2023b) used black phosphorus quantum dots as a tool to remove ROS, successfully alleviated ROS-induced damage, reduced MMP expression, and promoted NP regeneration. Shen et al. (2022) paid attention to the accumulation of lactic acid during the progression of IDD. They used lactate oxidase to lower the level of local lactic acid, thereby reducing the expression of proinflammatory factors, protecting cells from apoptosis, and enhancing the synthesis of ECM. Li et al. (2023a) tried to use human-derived platelet-rich plasma to treat IDD, prepared a nanofiber-reinforced injectable hydrogel, and successfully restored some functions of the IVD.

Furthermore, plasma-rich plasma has been used in some clinical scenarios. Kawabata et al. (2023) included two Modic type 1 LBP patients and administered intradiscal injection of plasma-rich plasma, which successfully improved their symptoms and suppressed inflammation. Meanwhile, according to reports, platelet-rich plasma injection therapy for lower back pain showed significant improvements in functional outcomes, pain relief, and patient satisfaction compared to placebo (intradiscal contrast agent). It is worth noting that patients who receive platelet-rich plasma injections can still alleviate pain and improve function even after several years of injection, while some surgical patients experience recurrent symptoms and are considered to have failed treatment (Machado et al., 2023).

### 3.4 Combined strategy

As previously stated, a range of bioactive substances, such as cells, genes, drugs, and others, have been employed in attempts to treat IDD. These substances exert their effects through distinct mechanisms of action, possess diverse action characteristics, and have yielded corresponding outcomes. Nevertheless, the utilization of a solitary bioactive substance for treatment often exhibits a unilateral and restricted approach. Consequently, numerous individuals have endeavored to combine various bioactive substances in order to attain enhanced and more comprehensive therapeutic effects. Kim et al. (2020) are based on human bone marrow-derived MSCs (BMSCs) and paired with TGF-β3. In this study, a conducive environment for the chondrogenic differentiation of BMSCs is created, leading to the effective induction of IVD regeneration in a dog model. Feng et al. (2020) successfully used rabbit MSCs with miRNA-199a to regenerate tissue resembling the NP *in vitro* and exhibit resistance to calcification *in vivo*, regenerating NP *in situ* in the animal lumbar spine. On the basis of BMSCs therapy, Sun et al. (2023) achieved successful protection of BMSCs from oxidative stress and facilitated their differentiation into phenotypes resembling the NP by employing a combination of coenzyme Q-10. Xu et al. (2020a) successfully used growth differentiation factor-5 to promote the differentiation of rat ADSCs, induce *in vitro* synthesis of NP-like coatings and ECM, and partially restore the degraded IVD through minimally invasive *in situ* injection. Yu et al. (2021) jointly delivered kartogenin (KGN) and apocynin (APO) into IVD, stimulating the differentiation of human ADSCs, enhancing their vitality, and resulting in heightened disc height and increased water content in rats.

Borrelli and Buckley (2020) used chondroitin sulfate to help nasal cartilage cells in maintaining a rounded cell morphology in 3D culture and to improve their capacity for synthesizing a matrix resembling the NP. Xia et al. (2023) combined ibuprofen and nucleus pulposus promoter cells (NPPCs), enabling it to better adapt to harsh IDD environments and improve its performance in IDD repair.

In addition to cell therapy, some studies have focused on the combination of gene and drug therapy. Wang et al. (2022b) locally and continuously delivered naturally extracted anti-inflammatory curcumin and antagomir-21 to treat IDD, successfully delaying the process of IDD and supporting IVD regeneration. Ding et al. (2022) compared Luteolin with TGF-β1 plasmid codelivery to NP and found that it significantly delayed rat tail IDD without significant local/systemic toxicity, demonstrating superior treatment strategies for IDD.

In summary, cell, gene, and drug strategies are currently popular methods for repairing degenerative IVD. These new treatment methods based on local injection of bioactive substances have less harm and a lower risk of sequelae and complications compared to traditional surgical treatments. They can accurately locate and achieve clearer therapeutic effects compared to conservative peripheral administration. More importantly, this therapy can target the core of the IDD process, inhibit the inflammatory microenvironment within the NP, reduce ROS generation, regulate ECM metabolism, and even reverse the IDD process. Nevertheless, owing to the intricate internal microenvironment of degenerative IVD, simple delivery systems generally have problems such as a short half-life, easy degradation, poor delivery efficiency, and high biological toxicity. Regardless of the treatment strategy, finding an intelligent and excellent delivery tool is particularly important. Therefore, we will review and summarize the existing delivery platforms.

## 4 Delivery platforms for therapeutic factors

As previously stated, cell therapy, gene therapy, drug therapy, and other bioactive substances have been explored as potential treatments for IDD. However, when these substances are directly injected into the IVD, their expulsion from the injection site is a common occurrence due to the substantial pressure applied on the IVD. In cases where drug release is not achieved, multiple injections within a short timeframe may be necessary, which has the potential to further undermine the mechanical durability of the IVD and exacerbate IDD. Consequently, the development of various biomaterial delivery systems, employing different sizes and based on different theoretical frameworks, has been pursued.

### 4.1 Nanocarriers

Nanocarriers are the most miniature delivery systems and are typically used to deliver small molecule drugs and small molecule genetic drugs. Nanocarriers prove effective in extending the retention time of drugs in the body, accomplishing the objective of prolonged drug loading and gradual release. In addition, nanocarriers can usually achieve lower biological toxicity and reduce unnecessary tissue damage.

#### 4.1.1 Extracellular vesicles

EVs are diminutive membranous vesicles released from cells into the extracellular matrix. They have the capability to stably transport crucial signaling molecules, playing an indispensable role in various physiological processes. Liao et al. (2019) used MSCs-derived EVs to successfully encourage the proliferation of NPCs and augment the generation of extracellular matrix *in vitro*, which has the capacity to alleviate apoptosis linked to endoplasmic reticulum stress in the context of IDD and potentially reverse the progression of IDD *in vivo*. Furthermore, they additionally illustrated that these EVs can proficiently shield NPCs from the repercussions of pyroptosis by delivering peroxiredoxin-2 and constructing an engineered EV that expresses caveolae-associated protein 2 in the EV membrane. Due to the repair of the endocytic pathway, it can restore the therapeutic effect in TNF-α-impaired NPCs (Liao et al., 2021b). In addition, they also found a role for metformin in overseeing both the quality and quantity of EVs derived from MSCs. Given the limitations of low production and unstable quality in the clinical use of EVs, this discovery may offer a more efficient approach for the treatment of IDD using EVs (Liao et al., 2021a).

#### 4.1.2 Nanomicelles

Micelles are structures with a core-shell arrangement, resulting from the self-assembly of amphiphilic copolymer molecules that possess both hydrophilic and hydrophobic ends. They typically have a hydrophobic core and hydrophilic shell surface, encapsulating hydrophobic drugs within them. According to the special microenvironment of IDD, different copolymer molecules can be designed to decompose under different conditions, achieving drug release.

Sun et al. (2023) directly used lecithin as a parental molecule to form micelles, encapsulating coenzyme Q-10 and combining with BMSCs, demonstrating good biosafety. Yu et al. (2021) connected the drug APO to the hydrophilic polyethylene glycol (PEG) chain through esterase-responsive phenol ester bonds to form an amphiphilic polymer micelle, which was loaded with the lipophilic drug KGN and used together with human ADSCs. *In vivo*, esterase-responsive phenolic ester bonds decompose in IVD, slowly releasing APO and KGN, which greatly improves the survival rate of human ADSCs and improves the therapeutic effect of IDD. Similarly, Xia et al. (2023) prepared the esterase-responsive ibuprofen copolymer poly (ethylene glycol)–poly [2-(methylacyloyl) ethylibuprofen] to form micelles, implanted it into NPPCs, and successfully made ibuprofen slow release in NPPCs, effectively avoiding the rapid degradation of ibuprofen *in vivo*.

In addition to the combination of carrying small molecule drugs and cell therapy, some studies directly use nano micelles to carry drugs to try to treat IDD. Feng et al. (2015) used the difference between human body temperature and external temperature to design a temperature-responsive nanomicelle using PEG-b-PASp (DET) and poly (N-isopropylacrylamide)-block-PASp(DET) [PNIPAM-b-PASp (DET)]. When the temperature of this micelle increases from room temperature (25°C) to body temperature (37°C), the PNIPAM segment can become insoluble and collapse on the surface of the micelle, making the pDNA carried inside it more difficult to degrade by nuclease, effectively improving the transfection efficiency of pDNA into cells. In another study, Tao et al. (2022) covalently bound hydrophobic palladium (II) meso-tetraphenyltetrabenzoporphyrin photocatalyst and coumarin-based NO donors with hydrophilic PEG to form nano micelles, which were relatively stable without premature NO release and achieved controlled NO release under *in vitro* red light irradiation catalysis. Ding et al. (2022) chose poly (e-caprolactone) and poly (β-amino ester synthesized block copolymers to form nano micelles, encapsulating Luteolin and pDNA for codelivery.

However, most of nanomicelles are still in the experimental stage within laboratories, and their capability as drug carriers falls short of fully meeting the clinical requirements for drug delivery. Secondly, an in-depth investigation is required to understand both the toxicological properties and the sustained release mechanisms associated with it.

#### 4.1.3 Other nanoparticles

In addition to EVs and nano micelles, there are some other nano micelle delivery systems, usually uniform spherical structures with various material sources, including polylactic acid, chitosan, and polydopamine. They have different physical and chemical characteristics and can also carry different drugs.

Lim et al. (2022) used poly (lactic-co-glycolic acid) nanoparticles to carry senolytic drug ABT263, achieving good biocompatibility, preventing the risk of potential systemic toxicity resulting from the systemic administration of inducers, as well as complications caused by repeated injection of free drugs into IVD, and successfully slowly releasing the drug. Teixeira et al. (2016) utilized Chitosan and Poly-γ-glutamic acid, which carry opposite charges and can self-assemble through electrostatic interactions. It carries diclofenac and delivers it into the IVD, prolonging its effect, controlling inflammation, and helping to reshape the ECM of the degraded IVD. Zhu et al. (2022) used hollow MnO_2_ to carry TGF-β3. It sustained release of TGF-β. At the same time, it exhibits sensitive pH responsiveness and H_2_O_2_ reactive oxygen generation, alleviating IVD hypoxia, reducing oxidative damage, and producing long-term therapeutic effects.

### 4.2 Microspheres

On the other hand, microparticles are micrometer-sized particles that are larger than nanoparticles and have a more complex structure. They can carry larger drugs, provide a platform for cell attachment, improve the success rate of cell delivery, and provide more complex release mechanisms to achieve more accurate and effective drug release.

**FIGURE 5:**
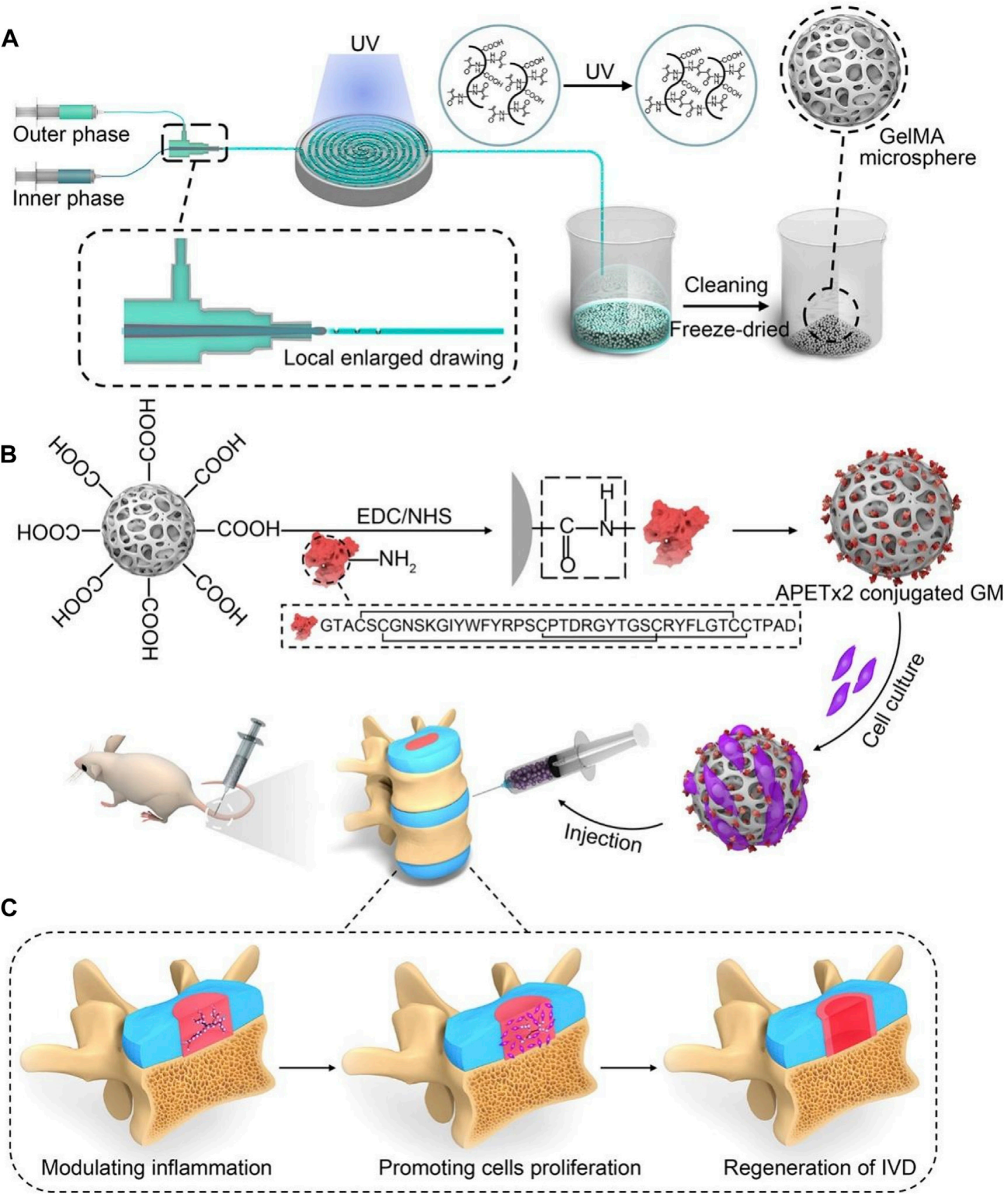
A schematic diagram illustrating the concept of injectable peptide-cell-hydrogel microspheres, designed to regulate local “inflammatory over-responses” and promote nucleus pulposus regeneration (Bian et al., 2021). **(A)** The production process of GelMA microspheres (GM) involves the use of a microfluidic synchronous cross-linking device. Following cleaning and freeze-drying, porous GM are prepared. **(B)** Preparing of APETx2 conjugated GelMA microspheres (GA) and cell-laden GA (GNA). And GNA is injected into the rat model of IVD degeneration. **(C)** The peptide-cell-hydrogel microspheres play a multifaceted role in the regeneration of IDD. This includes modulating local inflammation, promoting proliferation of nucleus pulposus cells, and contributing to the regeneration of the IVD.

GelMA is the most commonly used raw material for microspheres. For example, Xu et al. (2020a) successfully and rapidly manufactured GelMA microspheres with uniform size using the electroassisted bioprinting method, which were used to carry growth factors and rat ADSCs, providing substantial mechanical support while promoting cell adhesion, diffusion, and proliferation. Similarly, Bian et al. (2021) used microfluidics technology to construct GelMA microspheres, which were covalently coupled with APETx2 and further loaded with myeloid cells for the treatment of IDD and yielded positive outcomes (Figure 5). In addition, Kim et al. (2020) developed a leaf stack structural morphology that successfully and stably delivered human BMSCs and TGF-β3 to the IVD, which prevented cell leakage and provided a sustained supply of bioactive TGF-β3, inducing cell differentiation into cells resembling the NP. In another study, Chen et al. (2023c) used an air-microfluidic technique to prepare circadian clock-regulating microspheres made of polyvinyl alcohol, cross-linked them with modified phenylboronic acid (PBA) connectors and loaded them with liposomes containing melatonin.

### 4.3 Hydrogels

Hydrogels are structures that contain water and are formed by cross-linked natural or synthetic polymers, usually larger than particles. They can serve as delivery systems for small molecules, macromolecules, and cell therapy. More importantly, hydrogels are relatively fine particles, and their physical and chemical properties can be adjusted through composition and concentration to achieve appropriate drug delivery and provide mechanical properties that closely resemble those of the intervertebral disc (IVD), contributing to the better preservation of the IVD’s mechanical integrity. Through different gelation strategies, intelligent response to different microenvironments can also be achieved to achieve intelligent drug release, which has been achieved in many hydrogel materials for external wound dressings (Wang et al., 2021a; Wu et al., 2022b). Many materials have been used to construct hydrogels, encompassing natural derivatives like alginate, collagen, and HA, and synthetic materials like PEG.

#### 4.3.1 Alginate

Alginate brine gel stands out as one of the extensively utilized biocompatible materials. Therefore, it is also considered to be the basis of a potential delivery system that can be used to treat IDD. Based on alginic acid, Zeng et al. (2015) used poly (ethylene glycol) diacrylate (PEGDA) microcryogels to transform an alginic acid saline gel injection so that the alginate saline gel had a skeleton network and enhanced mechanical properties. With MSCs, they constructed a new injectable hydrogel, which successfully prevented cell leakage, enhanced cell retention and survival, and decreased NP denaturation in the model of IDD. Similarly, Tan et al. (2021) used Integrin and syndecan binding peptides (cyclic RGD and AG73, respectively) for the modification of acid salt gel and found that the modified Alginic acid salt gel increased cell attachment, vitality, biosynthetic activity, and expression of a NP-specific phenotype. In addition, Li et al. (2023a) mixed silk fibroin nanofibers into sodium alginate hydrogels, successfully enhancing the mechanical properties of sodium alginate hydrogels, showing excellent mechanical and leakproof properties, achieving the slow release of platelet-rich plasma, and providing valuable research for nanofiber-embedded hydrogels.

#### 4.3.2 Hyaluronic acid

Mohd Isa et al. (2018) demonstrated that HA hydrogel can reduce nociception and inhibit the expression of high innervation and nociceptive markers by changing glycosylation, weakening inflammatory signaling molecules, and regulating protein regulatory pathways. Furthermore, Chen et al. (2022) based on HA hydrogel, oxidized and synthesized aldehyde functionalized HA (HA-CHO), which was crosslinked with gene carrier Amine-terminated Generation 5 (G5) poly (amidoamine) (PAMAM) dendrimers carrying siRNAs to form a gel through dynamic Schiff base bond and successfully synthesized an injectable and self-healing hydrogel.

Besides, Chen et al. (2023a) used Girard reagent T-modified oxidized dextran (OG) and adipic acid dihydrazide (ADH)-grafted catechol-coupled gelatin (GCA) to crosslink through multiple dynamic bonds, including acylhydrazone bonds, imine bonds, hydrogen bonding between two interpenetrating polymer chains, and π - π stacking to form a gel. Furthermore, G5 PAMAM was used to carry siRNAs, successfully achieving sustained release of siRNAs for over 28 days. Yang et al. (2022) used OHA and borax to form a borate-diol complex and then further reacted it with gelatin through the Schiff base bond, carrying PBNPs, to produce a new type of double dynamic bond crosslinked injection self-healing hydrogel, which has excellent IDD repair mechanical properties and antibacterial and antioxidant properties.

HA methacrylate (HAMA) is a derivative of HA. Wang et al. (2022a) crosslinked it with methacrylated silk fibroin to form a hydrogel, matching the main matrix of AF and providing a suitable microenvironment for AF regeneration. Similarly, Huang et al. (2023) also used HAMA, which was crosslinked with GelMA, to deliver NP MSCs, providing mechanical properties and mechanical properties similar to those of NP tissue, improving the high osmotic pressure and low water content in the IDD microenvironment, and providing a specific bearing capacity.

#### 4.3.3 Gelatin

Gelatin is a product obtained from collagen denaturation. It is cheap, easy to obtain, and has good biocompatibility, so it is also a good natural hydrogel substrate. Wang et al. (2021b) prepared a gelatin gel hydrogel similar to natural NP. They carried MSCs, proving that this alternative has excellent biocompatibility, injectability, biodegradability, and the capability to facilitate MSCs differentiation into NP-like cells. Chen et al. (2023b) combined gelatin with alginic acid and used Zn^2+^ to promote cross-linking to prepare a robust biological hydrogel based on oxidized sodium alginate and gelatin. They delivered miR-204-3p to treat IDD, improving the mechanical properties of IVD and providing a favorable environment for genetic expression. To achieve better performance, Gan et al. (2017) designed a complex hydrogel system containing gelatin that incorporated gelatin with glucan as the primary network and gelatin with PEG as the secondary network to encapsulate NPCs. This hydrogel network shows excellent NP-like mechanical properties and toughness, reduces cell extrusion, facilitates prolonged cell survival and retention, and promotes the regeneration and rehydration of degenerative NP.

#### 4.3.4 Others

In addition to the above common hydrogel substrates, many other natural polymers are used for hydrogel construction. Self-assembling peptides are emerging biomedical materials polymerized from amino acids, possess excellent biocompatibility and high diversity, and can be modified according to different needs. Ligorio et al. (2021) used a self-assembled peptide FEFKFEFK (F: phenylalanine; K: lysine; E: glutamic acid) to construct a hydrogel and tried to treat IDD with GO-loaded TGF-β3, clearly indicating that GO reinforces the mechanical structure of self-assembled peptides and enables TGF-β3 slowly release. In another study, Tang et al. (2022) used self-assembled peptides as hydrogel substrates and directly modified bioactive polypeptide fragments on self-assembled peptides.

Synthetic polymers are also an excellent source of materials for building hydrogels. Synthetic materials are usually more able to meet our needs for hydrogels’ physical and chemical properties. However, their biocompatibility is generally lower than that of natural polymers, and the risk of adverse effects of degradation products on the human body is also greater. PEG is a kind of synthetic polymer commonly used to synthesize hydrogels. Chen et al. (2020) crosslinked 4-arm SH-PEG with Ag through Ag-S coordination and synthesized a multifunctional PEG hydrogel, which has injectable and self-healing properties, similar mechanical properties to normal IVD and good agomir loading capacity. Barcellona et al. (2021) developed a peptide-functionalized poly-PEG hydrogel system that uses laminin to mimic AG73 peptide and IKVAV functionalization, delivered NPCs, and maintained the viability of injected cells within 3 weeks. In addition, Huang et al. (2023) designed a poly (acrylamide coacrylic acid) microgel. Further, they coated it with dopamine to increase cell adhesion for the delivery of ADSCs, showing satisfactory cell adhesion and biocompatibility.

In general, hydrogels have a good application prospect in IDD treatment carriers due to their good biocompatibility, broad drug compatibility, and potential drug sustained-release activity and biomechanical properties. However, due to the large number of raw materials of hydrogels and the possibility of using various cross-linking agents in the gel-forming process, further research is still needed on whether there is more harm to the human body.

### 4.4 Combined strategy

Different delivery systems usually have different characteristics, and each has advantages and disadvantages. For example, micelles offer advantages in the delivery of hydrophobic drugs because of the presence of hydrophobic cores, while hydrogels are more suitable for the delivery of hydrophilic drugs due to their high water content.

**FIGURE 6:**
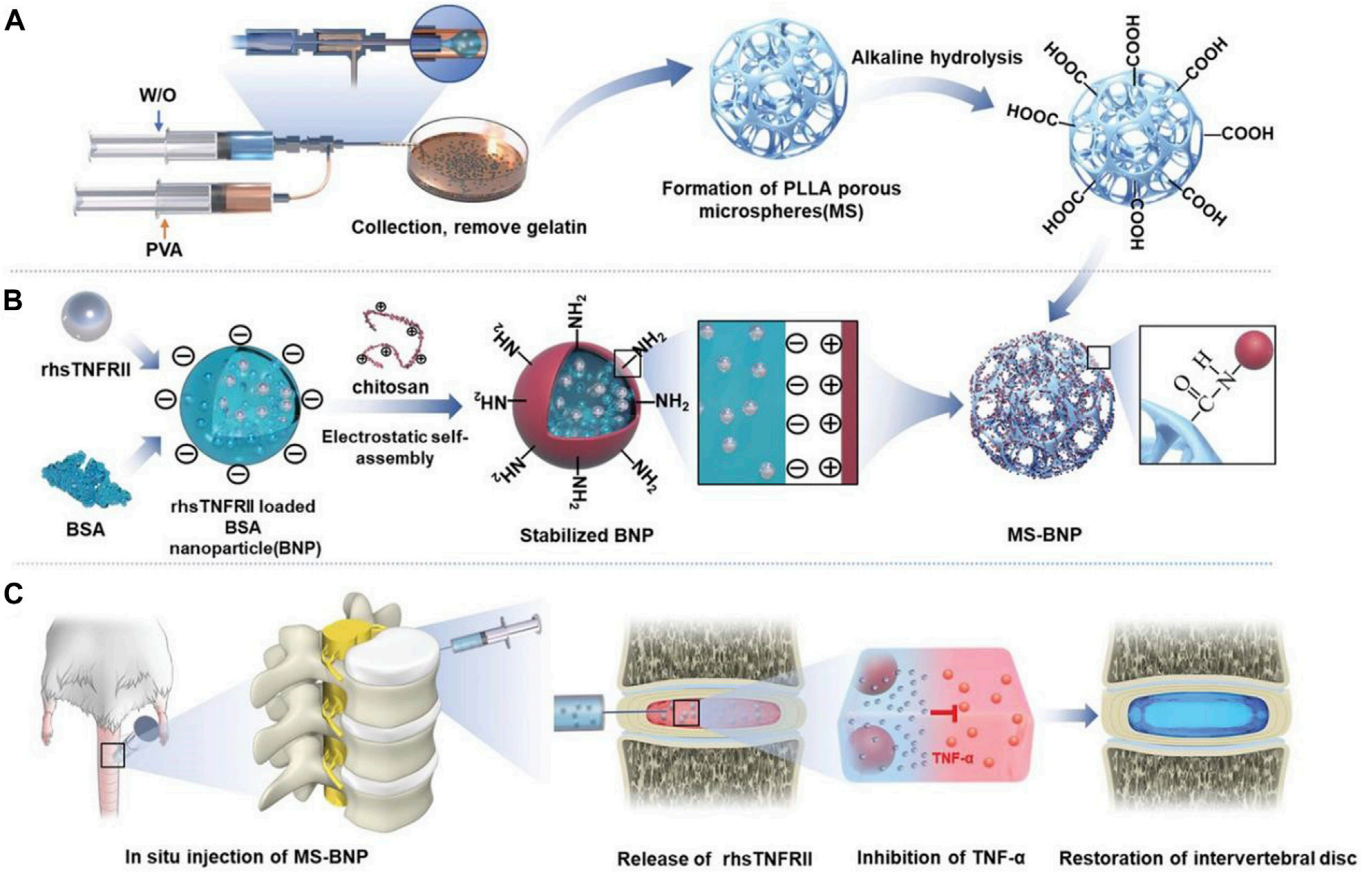
Schematic diagram of the structure and function of PLLA microspheres (Xu Y. et al., 2020b). **(A)** The preparation process and alkaline hydrolysis of PLLA microspheres. **(B)** The preparation process and transplantation of BNP loaded with rhsTNFRII. **(C)** Transplantation of MS-BNP.

In practical applications, multiple delivery systems of different sizes are often combined to achieve more controllable drug release and better therapeutic effects. Xu et al. (2020b) combined nanoparticles with microparticles and bovine serum albumin nanoparticles encapsulating rhsTNFRII were grafted onto microfluidic poly (l-lactic acid) (PLLA) porous microspheres through chemical bonds. It not only achieves uniform and continuous drug release but also buffers the compressed load through the mechanical strength of PLLA, providing a suitable environment for the recovery of IVD (Figure 6). Similarly, Chang et al. (2022) grafted DOTAP/Chol/DOPE cationic liposomes that encapsulate circSTC2 silencing genes onto HAMA microspheres through amide bonds to build “circRNA silencing hydrogel microspheres”. Shen et al. (2022) adsorbed lactate oxidase on MnO_2_ nanoparticles encapsulated in chitosan and then coupled this nanocomposite in large quantities to HAMA porous microspheres, significantly increasing enzyme loading and maintaining enzyme activity and concentration through covalent bonds, enduring regulation of the microenvironment over an extended period. Li et al. (2023b) grafted CS-containing phosphorus quantum dots onto the surface of GelMA porous microspheres, achieving a similar structure. Feng et al. (2017) first developed a hyperbranched polymer carrier wherein low molecular weight cationic polyethylenimine and short PEG chains were linked to the outer shell of the hydrophobic core of the molecule, successfully carrying NR4A1 pDNA. Then, they encapsulated it in biodegradable PLGA nanospheres and combined it with the newly developed nanofiber sponge microspheres to achieve a controlled release of pDNA. In another subsequent study, Feng et al. (2020) utilized this delivery strategy again and successfully equipped miR-199a to treat IDD.

Due to their large size, hydrogels can be easily combined with other small delivery systems and have excellent mechanical properties that are difficult for small delivery systems, which is usually an essential part of the combined delivery strategy. Various small delivery systems, such as exosomes and microspheres, have been used together with hydrogels and have achieved remarkable results. For example, Feng et al. (2018) developed an injectable hydrogel system cleavable by MMP to encapsulate miR-29a-loaded micelles responsive to MMP, achieving sustained and bioreactive two-stage miRNAs delivery. Similarly, Zheng et al. (2022) combined the hydrogel with liposomes. A composite hydrogel responsive to glucose, loaded with metformin-liposomes modified with phenylboronic acid, was developed. This not only achieves sustained drug release but also maintains the original mechanical strength of the IVD.

**FIGURE 7:**
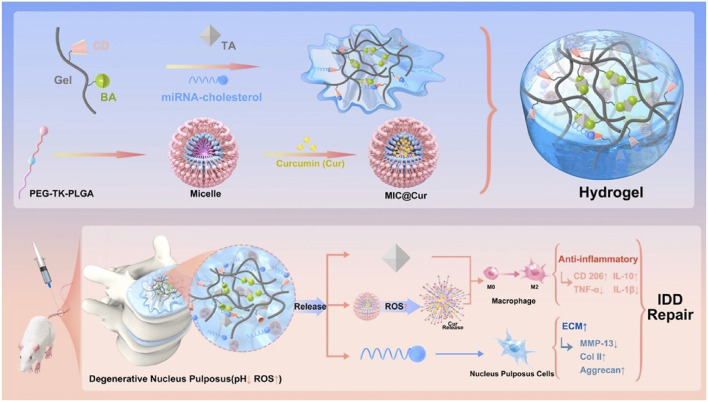
An illustration depicting the design of inflammation-responsive hydrogels, along with the mechanisms involved in promoting IDD repair (Wang Y. et al., 2022b). BA, phenylboronic acid; CD, cyclodextrin; Gel, gelatin; TA, tannic acid.

Wang et al. (2022b) grafted phenylboronic acid and cyclodextrin onto gelatin, which was used to cross-link with tannic acid and carry antagomiR-21, and then carried ROS-responsive amphiphilic polymer micelles coated with curcumin to construct an inflammatory reactive anti-inflammatory hydrogel (Figure 7). On that basis, Wang et al. (2023b) further developed a gene delivery system injectable with nanogel-encapsulated hydrogel, which has a three-stage miRNAs delivery mechanism. This system is also based on gelatin, which crosslinks 3,4,5-trihydroxybenzaldehyde-grafted gelatin and phenylboronic acid-grafted carboxymethyl chitosan through PEG dipropionate and loads dextran microgel carrying antagomiR-21 through cyclodextrin. In addition, Wang et al. (2023a) also used carboxymethyl chitosan gel to encapsulate the modified Tannic acid nanoparticles carrier to deliver antagomir-21 to the nucleus pulposus on demand and continuously. Panebianco et al. (2022) designed a complex multilevel delivery system. This system uses alginate oxide microbeads to wrap AF cells, forming a composite hydrogel with genipin-crosslinked fibrin. This hydrogel has higher biomechanical stability, promotes cell vitality and ECM synthesis enhancement, and will help IVD repair after injury.

In the clinical application of exosomes, the biggest challenge is to keep them stable in the body, and hydrogels can slow down the degradation of exosomes in the body. Xing et al. (2021) prepared a thermosensitive cell-free ECM hydrogel coupled with ADSCs exosomes to improve IDD. To further reduce the reduction in secretion *in vivo* and *in vitro*, Luo et al. (2022) injected an ECM hydrogel loaded with cartilage endplate stem cells that can produce exosomes *in vivo* near the CEP and finally improved IDD by producing Sphk2-engineered exosomes.

## 5 Conclusion and future perspectives

LBP caused by IDD is a public health issue of concern. However, there are still many problems with the treatment methods for LBP, and the conservative treatment effect is unclear. Surgical treatment also carries the risk of sequelae and complications, and both cannot be targeted at the core IDD of LPB, which cannot prevent the progression of IVD degeneration and can only alleviate the symptoms of LBP. Therefore, finding a method that can target the IDD process and fundamentally treat LBP is urgent.

The various therapeutic factors and corresponding local delivery systems introduced in this article may bring dawn to the precise treatment of LBP. These treatment methods are all based on precise local treatment of IDD, targeting changes in the metabolism, microenvironment, and even gene pathways of IDD, attempting to prevent the process of IDD and even reverse its degeneration. Traditional small molecule drugs may be more suitable as the earliest biologically active substances to enter clinical transformation due to their relatively complete theories and research. However, cellular and genetic strategies require further research due to their unclear mechanisms.

Various essential biomaterials are also used for the construction of delivery systems. Using different molecules and chemical modifications, we can also obtain various biomaterials with different properties to meet various requirements. For example, hydrogels formed based on reversible chemical bonds can spontaneously gel after injection, reducing tissue damage. Biodegradable biomaterials under different conditions can only correspond to the internal microenvironment of IDD, achieving precise drug release. Of course, these therapies are still far from clinical practice, and the effectiveness of bioactive substances and the biological safety of delivery systems still need to be verified.

In summary, an excellent IDD treatment system should meet the following requirements: 1) Targeting the core process of IDD can effectively prevent IVD degeneration. 2) It has good biological safety. 3) Capable of achieving slow, on-demand, and responsive drug release. 4) Having biomechanical properties similar to those of healthy IVDs, providing mechanical support for degenerative IVDs.
